# Supplementary material for: MYT1L deficiency impairs excitatory neuron trajectory during cortical development
Source: Nat Commun. 2024 Nov 27;15:10308. doi: 10.1038/s41467-024-54371-2 (PMC11603064; doi:10.1038/s41467-024-54371-2)
Supplement: Supplementary file 1 — Supplementary Information [file 41467_2024_54371_MOESM1_ESM.pdf]

Supplementary Materials for

MYT1L deficiency impairs excitatory neuron trajectory during cortical development

Allen Yen, Simona Sarafinovska, Xuhua Chen, Dominic D. Skinner, Fatjon Leti, MariaLynn Crosby, Jessica Hoisington-Lopez, Yizhe Wu, Jiayang Chen, Zipeng A. Li, Kevin K. Noguchi, Robi D. Mitra, Joseph D. Dougherty*

*Corresponding author. Email: jdougherty@wustl.edu (J.D.D.)

This PDF file includes:

Supplementary Figs. 1-5
Supplementary Table 1

Other supplementary material for this manuscript includes the following:

Supplementary Data 1-5 (tab-delimited format)

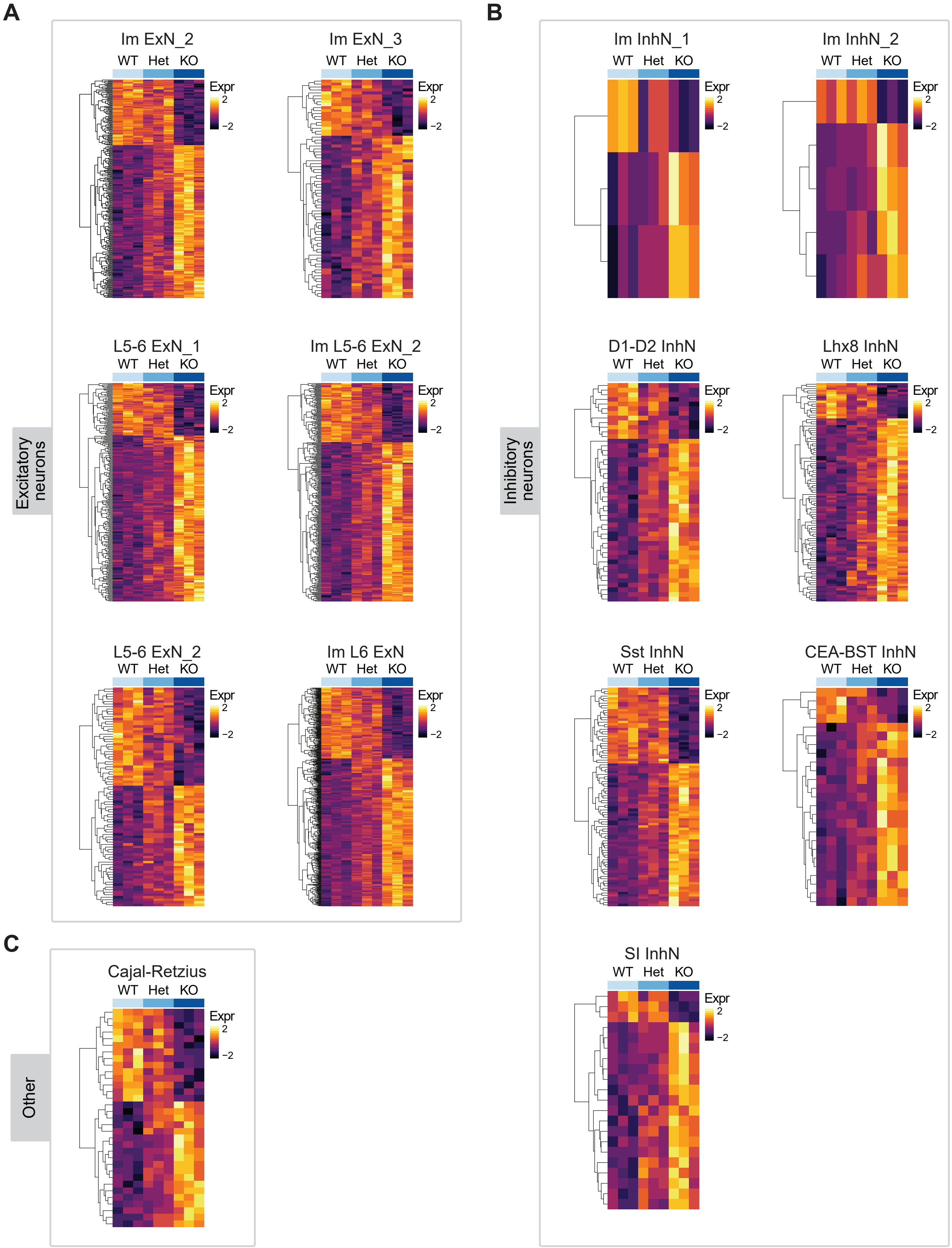

Supplementary Fig. 1. Gene dosage effects of differentially expressed genes per cluster at E14.
Heatmaps showing scaled gene expression per gene (row) for each genotype (columns) (n=3 biological replicates per genotype: WT, Het, and KO) for each cell type grouped by classes of (A) excitatory neurons, (B) inhibitory neurons, and (C) other. Source data can be found in Supplemental Table 3.

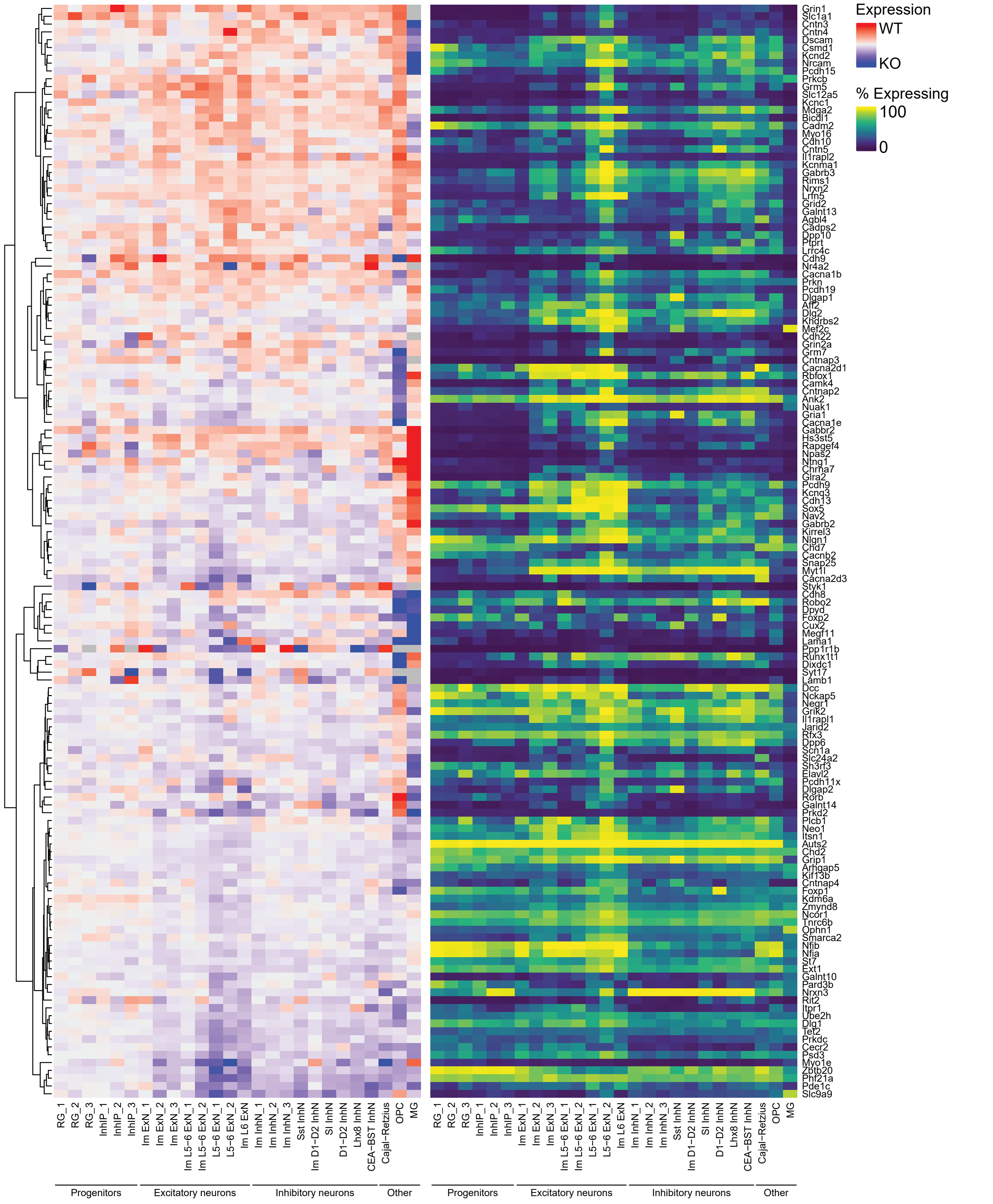

Supplemental Fig. 2. Differential expression of SFARI genes at E14.

The heatmap on the left shows scaled gene expression data of SFARI genes with a score of 1 or 2 (rows) per cluster (columns) that were found to be differentially upregulated in WT (red) or KO (blue) conditions. The heatmap on the right shows the percentage of cells in the same clusters that expressed each gene. Cell types are grouped into 4 main classes: progenitors, excitatory neurons, inhibitory neurons, and other.

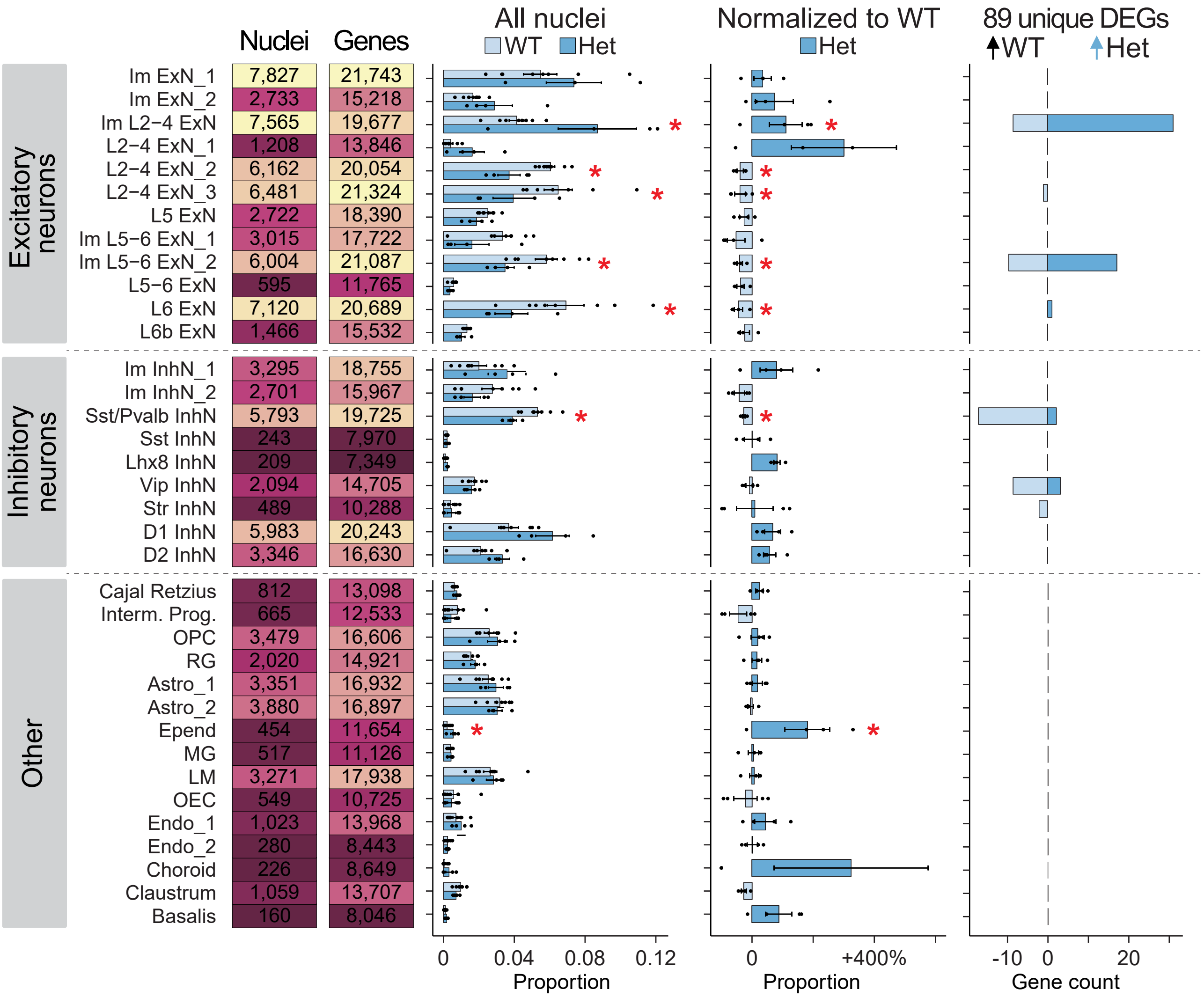

Supplementary Fig. 3. Single nucleus transcriptional profiling of P1 forebrain in MYT1L animals.
This figure shows all cell types from the P1 dataset. From left to right: summary plot showing the numbers of nuclei and genes detected in each cluster; bar plot displaying the mean±SEM relative proportions of nuclei in each annotated cell cluster for MYT1L WT and Het genotypes; mean±SEM proportions of Het normalized to WT; and the number of differentially expressed genes (DEGs) per cell type that are upregulated in WT (light blue; n=8 biological replicates) and upregulated in Het (medium blue; n=4 biological replicates) (*FDR adjusted p<0.05, moderated t-test).

A

B

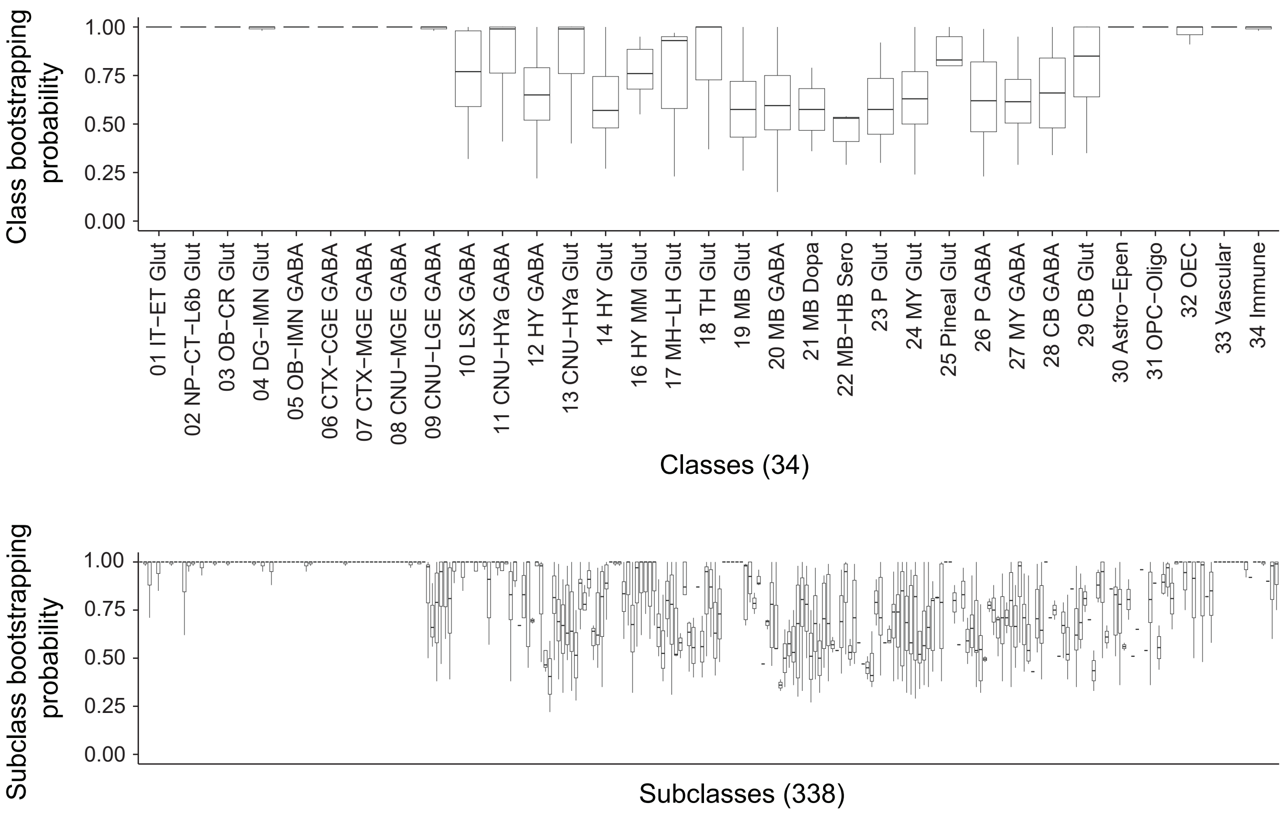

Supplementary Fig. 4. Standardized cell annotation of P21 dataset using the Allen Brain Cell Atlas.
(A) MapMyCells (RRID:SCR_024672) and heirarchical correlation mapping was used to align cells from this study to the Allen Brain Cell Atlas, consisting of ~4.3M cells annotated into 34 classes, 338 subclasses, 1201 supertypes, and 5322 clusters. For each cell, a random set of 90% of marker genes was selected, then mapped to the atlas by traversing the taxonomy by starting with classes, then proceeding to subclasses, supertypes, and clusters. This was repeated 100 times to obtain the boostratpping probability. The bootstrapping probability for each class is shown in (A) and subclass is shown in (B). Labels with a high bootstrapping probability (close to 1) are considered high confidence labels and subclass names were used to annotate nuclei from this study.

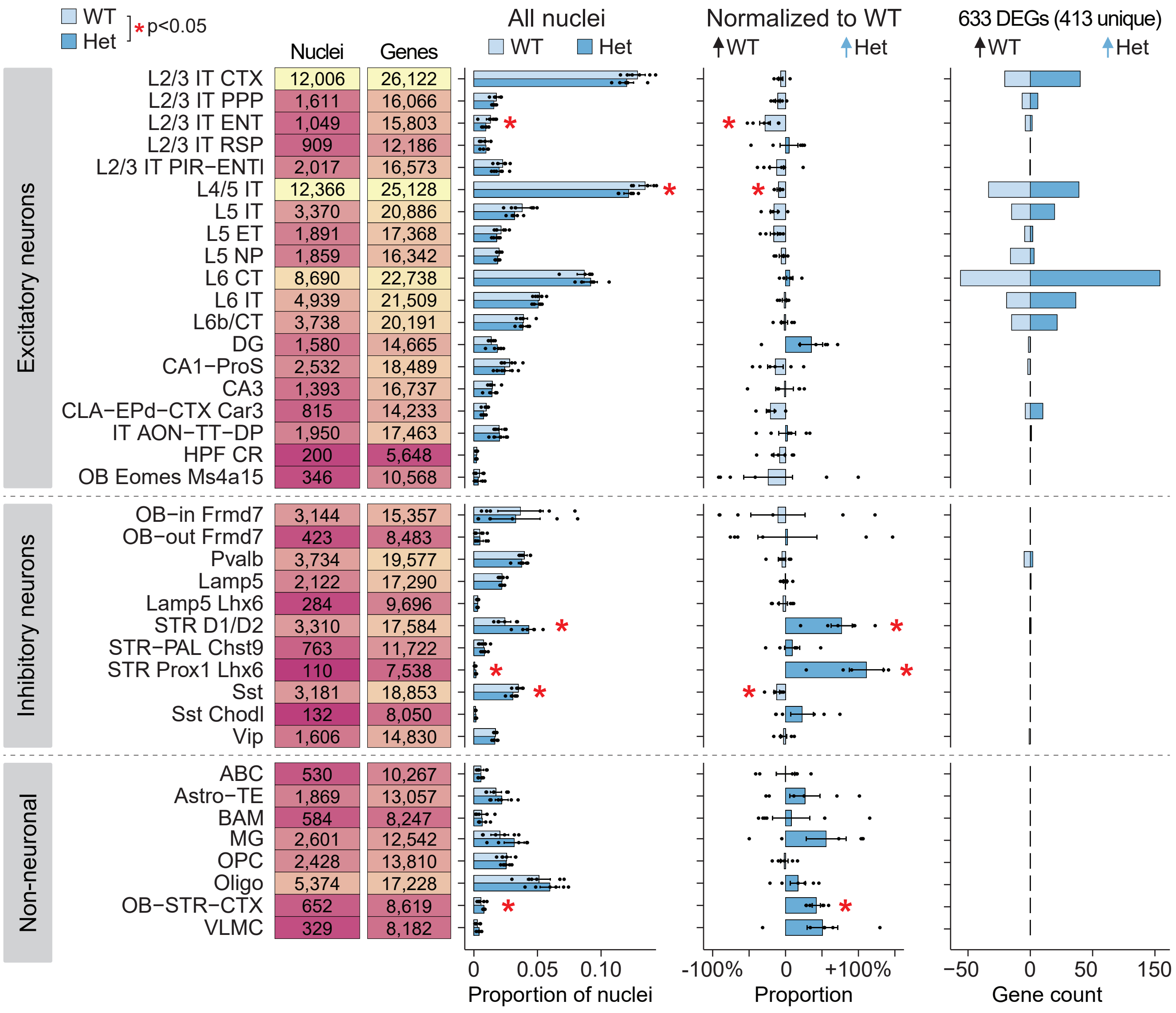

Supplementary Fig. 5. Single nucleus transcriptional profiling of P21 cortex in MYT1L animals.
This figure shows all cell types from the P21 dataset. From left to right: summary plot showing the numbers of nuclei and genes detected in each cluster; bar plot displaying the mean±SEM relative proportions of nuclei in each annotated cell cluster for MYT1L WT and Het genotypes; mean±SEM proportions of Het normalized to WT; and the number of differentially expressed genes (DEGs) per cell type that are upregulated in WT (light blue; n=6 biological replicates) and upregulated in Het (medium blue; n=6 biological replicates) (*FDR adjusted p<0.05, moderated t-test).

Study	Observation		Method, n
	**D1/D2 neurons**	**Ratio of Deep to Upper Layers**	
Chen et al. Neuron 2021	NA	No differences	Immunofluorescence, n=5
Chen et al. Genome Res 2023	NA	In mutants: more deep layer genes (E14), more deep layer genes (P21), trend towards less upper layer (P21)	Bulk RNAseq GSEA (Figure 1) of Neuron paper data
	NA	In mutants: more Bcl11b (L5/6 marker) density, no change in Pou3f2 (upper layer marker)	Immunohistochemistry, n=6
Weigel et al. Mol Psych 2023	In mutants: more D1/D2	In mutants: more Tbr1, fewer Tbr2, more Reln (L1)	P0 scRNAseq, n=2 per genotype
Yen et al. E14 dataset	In mutants: more D1/D2, but fewer immature D1/D2	In mutants: more Im ExN_3, fewer L5-6 ExN, fewer Im L6 ExN	E14 snRNAseq, n=3 WT, 3 Het, 3 KO
Yen et al. P1 dataset	In mutants: more D1/D2, but not significant	In mutants: more Im L2-4 type 1, fewer Im L2-4 type 2, fewer L5-6 ExN, fewer L6 ExN	P1 snRNAseq, n=8 WT, 4 Het
Yen et al. P21 dataset	In mutants: more D1/D2	In mutants: fewer L2/3 IT ENT, more L2/3 IT PIR-ENTI but not significant, fewer L4/5 IT, more L6 CT, more L6 IT, more L6b CT	P21 snRNAseq, n=6 WT, 6 Het
	In mutants: more Darpp32+ neurons in cortex		Immunofluorescence, n=9 WT, 9 Het

Supplementary Table 1. Comparison of neuronal layer distribution across multiple studies.
This table summarizes observations from various studies examining the effects of MYT1L deficiency on neuronal layer distribution in the cortex and D1/D2 proportions across different developmental stages and various experimental methods.
