# Supplementary material for: MYT1L deficiency impairs excitatory neuron trajectory during cortical development
Source: Nat Commun. 2024 Nov 27;15:10308. doi: 10.1038/s41467-024-54371-2 (PMC11603064; doi:10.1038/s41467-024-54371-2)
Supplement: Supplementary file 2 — Description of Additional Supplementary Files [file 41467_2024_54371_MOESM2_ESM.pdf]

Description of Additional Supplementary Files

File Name: Supplementary Data 1
Description: Differentially expressed genes (DEGs) comparing wild-type (WT) and knockout (KO) MYT1L in E14 nuclei, presented as pseudobulk data in tab-delimited format. Negative log2FoldChange values indicate genes upregulated in WT, while positive values indicate genes upregulated in KO.

File Name: Supplementary Data 2
Description: Differentially expressed genes (DEGs) comparing wild-type (WT) and heterozygous (Het) MYT1L in E14 nuclei, presented as pseudobulk data in tab-delimited format. Negative log2FoldChange values indicate genes upregulated in WT, while positive values indicate genes upregulated in Het.

File Name: Supplementary Data 3
Description: Dose-dependent differentially expressed genes (DEGs) in response to MYT1L levels in E14 nuclei, presented as pseudobulk data in tab-delimited format.

File Name: Supplementary Data 4
Description: Differentially expressed genes (DEGs) comparing wild-type (WT) and heterozygous (Het) MYT1L in P1 nuclei, presented as pseudobulk data in tab-delimited format. Negative log2FoldChange values indicate genes upregulated in WT, while positive values indicate genes upregulated in Het.

File Name: Supplementary Data 5
Description: Differentially expressed genes (DEGs) comparing wild-type (WT) and heterozygous (Het) MYT1L in P21 nuclei, presented as pseudobulk data in tab-delimited format. Negative log2FoldChange values indicate genes upregulated in WT, while positive values indicate genes upregulated in Het.
