# Supplementary material for: MYT1L deficiency impairs excitatory neuron trajectory during cortical development
Source: Nat Commun. 2024 Nov 27;15:10308. doi: 10.1038/s41467-024-54371-2 (PMC11603064; doi:10.1038/s41467-024-54371-2)
Supplement: Supplementary file 8 — Reporting Summary [file 41467_2024_54371_MOESM8_ESM.pdf]

nature portfolio

Corresponding author(s): Joseph D. Dougherty

Last updated by author(s): Sep 4, 2024

Reporting Summary

Nature Portfolio wishes to improve the reproducibility of the work that we publish. This form provides structure for consistency and transparency in reporting. For further information on Nature Portfolio policies, see our Editorial Policies and the Editorial Policy Checklist.

Statistics

For all statistical analyses, confirm that the following items are present in the figure legend, table legend, main text, or Methods section.

n/a	Confirmed	
☐	☒	The exact sample size (n) for each experimental group/condition, given as a discrete number and unit of measurement
☐	☒	A statement on whether measurements were taken from distinct samples or whether the same sample was measured repeatedly
☐	☒	The statistical test(s) used AND whether they are one- or two-sided<br>*Only common tests should be described solely by name; describe more complex techniques in the Methods section.*
☒	☐	A description of all covariates tested
☐	☒	A description of any assumptions or corrections, such as tests of normality and adjustment for multiple comparisons
☐	☒	A full description of the statistical parameters including central tendency (e.g. means) or other basic estimates (e.g. regression coefficient) AND variation (e.g. standard deviation) or associated estimates of uncertainty (e.g. confidence intervals)
☐	☒	For null hypothesis testing, the test statistic (e.g. F, t, r) with confidence intervals, effect sizes, degrees of freedom and P value noted<br>*Give P values as exact values whenever suitable.*
☒	☐	For Bayesian analysis, information on the choice of priors and Markov chain Monte Carlo settings
☒	☐	For hierarchical and complex designs, identification of the appropriate level for tests and full reporting of outcomes
☒	☐	Estimates of effect sizes (e.g. Cohen's d, Pearson's r), indicating how they were calculated

Our web collection on statistics for biologists contains articles on many of the points above.

Software and code

Policy information about availability of computer code

Data collection	NovaSeqX Plus onboard DRAGEN was used to run BCL Convert v4.1.23 to generate demultiplexed files from an Illumina run as input. Microscopy images were collected using ZEN 3.9.
Data analysis	The ScaleBio Single-Cell RNA Workflow v1.4 (https://github.com/ScaleBio/ScaleRna) was used to generate feature-barcode matrices from fastq files. CellFinder from ScaleRna v1.5 was used for ambient RNA removal and enhanced recovery of nuclei with low total RNA content. The following software was used: Nextflow v23.04.4, SingularityCE v3.10.3. R v4.2.2 packages Seurat v4.3.0, DoubletFinder v2.0.3, DESeq2 v1.38.3, ageglm v1.20.0, monocle3 v1.3.1. Python 3.9.18 packages scanpy v1.12.0 and pySCENIC v0.12.1 were used. Custom code is available at bitbucket (https://bitbucket.org/jdlabteam/yen-et-al-myt1l-snrnaseq/src/main/). Image processing and analysis was performed using ImageJ v1.54f and R package lme4 v1.1-35.5.

For manuscripts utilizing custom algorithms or software that are central to the research but not yet described in published literature, software must be made available to editors and reviewers. We strongly encourage code deposition in a community repository (e.g. GitHub). See the Nature Portfolio guidelines for submitting code & software for further information.

Data

Policy information about availability of data

All manuscripts must include a data availability statement. This statement should provide the following information, where applicable:

- Accession codes, unique identifiers, or web links for publicly available datasets
- A description of any restrictions on data availability
- For clinical datasets or third party data, please ensure that the statement adheres to our policy

Raw and analyzed sequencing data are available at Gene Expression Omnibus (GEO; SuperSeries GSE262368).

Research involving human participants, their data, or biological material

Policy information about studies with human participants or human data. See also policy information about sex, gender (identity/presentation), and sexual orientation and race, ethnicity and racism.

Reporting on sex and gender	Human research was not conducted in this study.
Reporting on race, ethnicity, or other socially relevant groupings	Human research was not conducted in this study.
Population characteristics	Human research was not conducted in this study.
Recruitment	Human research was not conducted in this study.
Ethics oversight	Human research was not conducted in this study.

Note that full information on the approval of the study protocol must also be provided in the manuscript.

Field-specific reporting

Please select the one below that is the best fit for your research. If you are not sure, read the appropriate sections before making your selection.

☒ Life sciences ☐ Behavioural & social sciences ☐ Ecological, evolutionary & environmental sciences

For a reference copy of the document with all sections, see nature.com/documents/nr-reporting-summary-flat.pdf

Life sciences study design

All studies must disclose on these points even when the disclosure is negative.

Sample size	No statistical methods were used to predetermine sample sizes for snRNAseq. Sample sizes were selected to balance assaying an adequate number of nuclei from each animal with biological replicates. For E14 snRNAseq samples, we used 3 biological replicates per genotype (WT, Het, and KO) with mixed sexes, totally 9 samples. The P1 cohort comprised 12 animals with 8 WT and 4 Het. The P21 cohort included 12 animals: 3 male WT, 3 female WT, 3 male MYT1L Het, and 3 female MYT1L Het. These sample size were deemed sufficient to ensure reliable detection of genotype-specific responses while balancing the need for reproducible data with practical constraints of the experimental design. For immunofluorescence analysis, sample size was established based on prior similar studies. All replicates (9 WT and 9 Het) were included and analyzed.
Data exclusions	No animal samples were excluded from the analyses. For the snRNAseq data analysis, cell barcodes associated with ambient RNA was removed using CellFinder. Doublets and multiplets were excluded using DoubletFinder. Barcodes that met any of the following criteria were filtered out: 1) <800 UMIs; 2) >6000 UMIs; 3) <300 genes; 4) >3000 genes; 5) % mitochondrial reads > 1%; 6) log(genes)/log(UMIs) < 0.9.
Replication	The snRNAseq assays were only run once per E14, P1, and P21 age groups and thus not replicated. However, incorporating multiple biological replicates within groups enhances the robustness of key findings by accounting for variation between biological replicates, thus minimizing false discoveries.
Randomization	The samples for snRNAseq were randomly selected from available specimens across 3 litters of E14 animals, 4 litters of P1 animals, and 4 litters of P21 animals that have been grouped by genotype.
Blinding	Blinding was not used for snRNAseq library preparation since we needed to determine which samples to process and load into barcoded sample wells for proper demultiplexing. For the computational analysis, all samples were analyzed using the same code and parameters. For analysis of immunofluorescence data, the investigator was blinded during image processing and counting of double-positive DARPP32 and NeuN nuclei.

Reporting for specific materials, systems and methods

We require information from authors about some types of materials, experimental systems and methods used in many studies. Here, indicate whether each material, system or method listed is relevant to your study. If you are not sure if a list item applies to your research, read the appropriate section before selecting a response.

Materials & experimental systems

n/a	Involved in the study
☐	☒ Antibodies
☒	☐ Eukaryotic cell lines
☒	☐ Palaeontology and archaeology
☐	☒ Animals and other organisms
☒	☐ Clinical data
☒	☐ Dual use research of concern
☒	☐ Plants

Methods

n/a	Involved in the study
☒	☐ ChIP-seq
☒	☐ Flow cytometry
☒	☐ MRI-based neuroimaging

Antibodies

Antibodies used

The following primary antibodies were used: recombinant rabbit monoclonal anti-DARPP32 clone SU0329 (1:200, Invitrogen MA5-32113) and mouse monoclonal anti-NeuN clone 1B7 (1:400, Invitrogen MA5-33103). The following secondary antibodies were used: donkey anti-rabbit Alexa Fluor 488 (1:400, Invitrogen A-21206) and donkey anti-mouse Alexa Fluor 568 (1:400, Invitrogen A10037).

Validation

Primary antibodies were titrated to determine the dilution that provides optimal signal with minimal background fluorescence. To assess background fluorescence and autofluorescence levels, we performed control stainings omitting the primary antibodies.
The rabbit monoclonal anti-DARPP32 antibody (Invitrogen MA5-32113) has 1 published citation. Kraskovskaya et al. (Int. J. Mol. Sci. 2023) used this antibody to identify DARPP32 expression in induced medium spiny neurons that were directly reprogrammed from human fibroblasts. In our own immunofluorescence staining of brain sections, the antibody revealed highest expression and localization in the striatum, which aligns with the expected expression profile of DARPP32 as documented in the in situ hybridization (ISH) Atlas from the Allen Institute.
The mouse monoclonal anti-NeuN antibody (Invitrogen MA5-32113) has 7 published citations listed on the manufacturer's website.
For detection, we used donkey secondary antibodies conjugated to Alexa Fluor dyes. These secondary antibodies are widely adopted in the scientific community. On the manufacturer's website, the donkey anti-rabbit Alexa Fluor 488 has 6,891 references and the donkey anti-mouse Alexa Fluor 568 has 985 references.

Animals and other research organisms

Policy information about studies involving animals; ARRIVE guidelines recommended for reporting animal research, and Sex and Gender in Research

Laboratory animals

All animal studies were approved by and performed in accordance with the guidelines of the Animal Care and Use Committee of Washington University in Saint Louis, School of Medicine (Protocol 23-0138) and conformed to NIH guidelines for the care and use of laboratory animals. Animals were housed in controlled environments with a 12-hour light-dark cycle, constant temperature and relative humidity, and ad libitum access to food and water. The C57BL/6-Myt1lem1Jdd/J (Myt1l S710fsX12; Jackson Laboratories 036428) line was maintained with breeding pairs consisting of a Myt1l Het and an in-house C57BL/6J mouse. The transgenic line was refreshed every 8-10 generations by backcrossing to freshly obtained C57BL/6J males and females from Jackson Laboratories. Upon weaning at P21, the animals were group-housed by sex and genotype. To obtain homozygous embryos, timed pregnant Myt1l Het x Het breeding pairs were set up, with the first day after finding a vaginal plug considered E0.5.
E14-14.5 embryos were rapidly dissected in HBSS on ice. Pups were decapitated, and the brains were quickly extracted, meninges removed, forebrains dissected, flash frozen in liquid nitrogen, and stored at -80°C. Tail tissue was collected for gDNA isolation and genotyping. The forebrains from P1 pups and cortical tissue from P21 pups were similarly collected and stored at -80°C. For the E14 cohort, mixed sexes from 3 biological replicates per WT, Het, and KO genotypes were used, totaling 9 samples. The P1 and P21 cohorts included only WT and Het animals, as KOs are not viable postnatally. The P1 cohort consisted of 12 animals, with 8 WT (5 male, 3 female) and 4 MYT1L Het (3 male, 1 female). The P21 cohort consisted of 12 animals: 3 male WT, 3 female WT, 3 male MYT1L Het, and 3 female MYT1L Het.

Wild animals

No wild animals were used in this study.

Reporting on sex

Both male and female mice were used for all experiments within this study. For E14 data, samples per genotype were mixed males and females and not powered to detect effects of sex. For P1, we had 5 male WT, 3 female WT, 3 male Het, and 1 female Het. For P21, we had 3 males and 3 females per genotype. We did not find any differential sex x genotype interactions likely because we were not powered to detect the subtle effects of sex.

Field-collected samples

No field-collected samples were used in this study.

Ethics oversight	All procedures involving animals were approved by the Washington University Institutional Animal Care and Use Committee (Protocol #23-0138).

Note that full information on the approval of the study protocol must also be provided in the manuscript.

Plants

Seed stocks	*Report on the source of all seed stocks or other plant material used. If applicable, state the seed stock centre and catalogue number. If plant specimens were collected from the field, describe the collection location, date and sampling procedures.*
Novel plant genotypes	*Describe the methods by which all novel plant genotypes were produced. This includes those generated by transgenic approaches, gene editing, chemical/radiation-based mutagenesis and hybridization. For transgenic lines, describe the transformation method, the number of independent lines analyzed and the generation upon which experiments were performed. For gene-edited lines, describe the editor used, the endogenous sequence targeted for editing, the targeting guide RNA sequence (if applicable) and how the editor was applied.*
Authentication	*Describe any authentication procedures for each seed stock used or novel genotype generated. Describe any experiments used to assess the effect of a mutation and, where applicable, how potential secondary effects (e.g. second site T-DNA insertions, mosiacism, off-target gene editing) were examined.*
